# Supplementary material for: Xolotl: An Intuitive and Approachable Neuron and Network Simulator for Research and Teaching
Source: Front Neuroinform. 2018 Nov 26;12:87. doi: 10.3389/fninf.2018.00087 (PMC6275287; doi:10.3389/fninf.2018.00087)
Supplement: Supplementary file 2 [file Data_Sheet_1.PDF]

Supplementary Material: Xolotl: An Intuitive and Approachable Neuron and Network Simulator for Research and Teaching

1 DATABASE OF CONDUCTANCES

Dynamics for model conductances in the following papers have been implemented in `xolotl`.

1. EAGes, EAGmut, EAGwt (Bronk et al. 2018)
2. A-type (Brookings, Goeritz, and Marder 2014)
3. Proctolin modulatory input current (Caplan, Williams, and Marder 2014)
4. CaT, H, Kd, NaV (Dethier, Drion, and Sepulchre 2015)
5. CaL, CaPump, KCa, Kd, NaV (Drion 2017)
6. Ap, At, Cal, KCa, KCaT, Proc (Goldman et al. 2001)
7. Shaker (Hardie et al. 1991)
8. A novel potassium conductance, Shab, Shaker (Heras, Vähäsöyrinki, and Niven 2018)
9. A-type, CaS, CaT, H, KCa, Kd, NaV (Kispersky, Caplan, and Marder 2012)
10. NaP, NaT (Lin et al. 2012)
11. A-type, CaS, CaT, H, KCa, Kd, NaV (Liu et al. 1998)
12. Liu et al. 1998 conductances with cached gating functions
13. Liu et al. 1998 conductances with forward Euler integration
14. Liu et al. 1998 conductances with temperature dependence (*cf.* Caplan, Williams, and Marder 2014)
15. Kd and NaV conductances from Int1, LG, and MCN1 cells (Nadim, Manor, Nusbaum, et al. 1998)
16. Calcium current (Nadim, Manor, Kopell, et al. 1999)
17. Drosophila NaV (O'Dowd and Aldrich 1988)
18. A-type, CaS, CaT, H, KCa, Kd, NaV (Prinz, Billimoria, and Marder 2003)
19. Prinz, Billimoria, and Marder 2003 conductances with cached gating functions
20. Prinz, Billimoria, and Marder 2003 conductances with temperature dependence (*cf.* Caplan, Williams, and Marder 2014)

21. Kd and NaV from Int1 cells and modulatory input and transient LTS conductances from LG cells (Rodriguez, Blitz, and Nusbaum 2013)
22. Proctolin modulatory input (Sharp et al. 1993)
23. Cal and Kd from AB-PD, LP, and PY cells (Soto-Treviño, Thoroughman, et al. 2001)
24. A-type, CaT, and KCa from AB and PD cells, generic H, Kd, NaP, NaV, and proctolin conductances (Soto-Treviño, Rabbah, et al. 2005)
25. Proctolin modulatory input (Swensen and Marder 2001)
26. A-type, CaS, CaT, H, KCa, Kd, NaP, NaV (Turrigiano, LeMasson, and Marder 1995)
27. Drosophila NaV (Wicher, Walther, and Wicher 2001)

2 PARAMETERS FOR SIMULATIONS

Two single-compartment models were simulated in this paper. The first, a Hodgkin-Huxley model with three conductances (`NaV`, `Kd`, and `Leak`) and injected current was simulated for Figure 1 and 7. The second is a stomatogastric neuron model with eight conductances (`NaV`, `CaT`, `CaS`, `ACurrent`, `KCa`, `Kd`, `HCurrent`, `Leak`) and was simulated for Figure 3 and 7. Both models have dynamics as described in Liu et al. 1998.

2.1 Parameters for Hodgkin-Huxley model

Parameter Name	Value	Units
Membrane capacitance (`HH.Cm`)	10	nF/mm^2
Surface area (`HH.A`)	0.01	mm^2
Fast sodium maximal conductance (`HH.NaV.gbar`)	1000	$\mu S/mm^2$
Delayed rectifier maximal conductance (`HH.Kd.gbar`)	300	$\mu S/mm^2$
Passive leak maximal conductance (`HH.Leak.gbar`)	1	$\mu S/mm^2$
Sodium reversal potential (`HH.NaV.E`)	50	mV
Potassium reversal potential (`HH.Kd.E`)	-80	mV
Leak reversal potential (`HH.Leak.E`)	-50	mV
Injected current (`I_ext`)	0.2	nA

2.2 Parameters for Stomatogastric Model

Parameter Name	Value	Units
Membrane capacitance (AB.Cm)	10	nF/mm^2
Surface area (AB.A)	0.0628	mm^2
Calcium buffering ϕ (AB.phi)	90	$\mu M/nA$
Calcium buffering shell volume (AB.vol)	0.0628	mm^3
Fast sodium maximal conductance (AB.NaV.gbar)	1831.2	$\mu S/mm^2$
Transient calcium maximal conductance (AB.CaT.gbar)	22.93	$\mu S/mm^2$
Slow calcium maximal conductance (AB.CaS.gbar)	27.07	$\mu S/mm^2$
Transient potassium maximal conductance (AB.ACurrent.gbar)	246.02	$\mu S/mm^2$
Calcium-gated potassium maximal conductance (AB.KCa.gbar)	979.94	$\mu S/mm^2$
Delayed rectifier maximal conductance (AB.Kd.gbar)	610.03	$\mu S/mm^2$
Hyperpolarization-activated maximal conductance (AB.HCurrent.gbar)	10.1	$\mu S/mm^2$
Passive leak maximal conductance (AB.Leak.gbar)	0.99045	$\mu S/mm^2$
Sodium reversal potential (AB.NaV.E)	30	mV
Potassium reversal potential (AB.Kd.E)	-80	mV
Hyperpolarization-activated reversal potential (AB.HCurrent.E)	-20	mV
Leak reversal potential (AB.Leak.E)	-50	mV

2.3 Parameters for Network Model

The network model displayed in Figure 4 is described in Prinz, Billimoria, and Marder 2003; Prinz, Bucher, and Marder 2004. It is comprised of three compartments AB, LP, and PY with the same dynamics but differing parameters and synaptic inputs.

Parameter Name	AB	LP	PY	Units
Fast sodium maximal conductance	1000	1000	1000	$\mu S/mm^2$
Transient calcium maximal conductance	25	0	24	$\mu S/mm^2$
Slow calcium maximal conductance	60	40	20	$\mu S/mm^2$
Transient calcium maximal conductance	500	200	500	$\mu S/mm^2$
Calcium-gated potassium maximal conductance	50	0	0	$\mu S/mm^2$
Delayed rectifier maximal conductance	1000	250	1250	$\mu S/mm^2$
Hyperpolarization-activated maximal conductance	0.1	0.5	0.5	$\mu S/mm^2$
Leak maximal conductance	0	0.3	0.1	$\mu S/mm^2$
Sodium reversal potential	50	50	50	mV
Potassium reversal potential	-80	-80	-80	mV
Hyperpolarization-activated reversal potential	-20	-20	-20	mV
Leak reversal potential	-50	-50	-50	mV

There are seven synapses in the model of two types: glutamatergic (Glut) and cholinergic (Chol) with differing dynamics. The synapse produces a current in the postsynaptic compartment based on the membrane potential in the presynaptic compartment.

**Presynaptic**	**Postsynaptic**	**Type**	$\bar{g}$ ($\mu S/mm^2$)
AB	LP	Chol	30
AB	PY	Chol	3
AB	LP	Glut	30
AB	PY	Glut	10
LP	PY	Glut	1
PY	LP	Glut	30
LP	AB	Glut	30

REFERENCES

Bronk, Peter, Elena A. Kuklin, Srinivas Gorur-Shandilya, Chang Liu, Timothy D. Wiggin, Martha L. Reed, et al. (2018) “Regulation of Eag by Ca2+/Calmodulin Controls Presynaptic Excitability in Drosophila”. In: *Journal of Neurophysiology* 119.5, pp. 1665–1680. DOI: 10.1152/jn.00820.2017.

Brookings, Ted, Marie L. Goeritz, and Eve Marder (2014) “Automatic Parameter Estimation of Multicompartmental Neuron Models via Minimization of Trace Error with Control Adjustment”. In: *Journal of Neurophysiology* 112.9, pp. 2332–2348. DOI: 10.1152/jn.00007.2014.

Caplan, Jonathan S., Alex H. Williams, and Eve Marder (2014) “Many Parameter Sets in a Multicompartment Model Oscillator Are Robust to Temperature Perturbations”. In: *Journal of Neuroscience* 34.14, pp. 4963–4975. DOI: 10.1523/JNEUROSCI.0280-14.2014.

Dethier, Julie, Guillaume Drion, and Rodolphe Sepulchre (2015) “A Positive Feedback at the Cellular Level Promotes Robustness and Modulation at the Circuit Level”. In: *Journal of Neurophysiology* 114.4. In collab. with Alessio Franci, pp. 2472–2484. DOI: 10.1152/jn.00471.2015.

Drion, Guillaume (2017) *The Dopamine Pacemaker Neuron Model*. Assignment. University of Rhode Island.

Goldman, Mark S., Jorge Golowasch, Eve Marder, and L. F. Abbott (2001) “Global Structure, Robustness, and Modulation of Neuronal Models”. In: *Journal of Neuroscience* 21.14, pp. 5229–5238. DOI: 10.1523/JNEUROSCI.21-14-05229.2001.

Hardie, Roger C., Dieter Voss, Olaf Pongs, and Simon B. Laughlin (1991) “Novel Potassium Channels Encoded by the Shaker Locus in Drosophila Photoreceptors”. In: *Neuron* 6.3, pp. 477–486. DOI: 10.1016/0896-6273(91)90255-X.

Heras, Francisco J. H., Mikko Vähäsöyrinki, and Jeremy E. Niven (2018) “Modulation of Voltage-Dependent K+ Conductances in Photoreceptors Trades off Investment in Contrast Gain for Bandwidth”. In: *bioRxiv*, p. 344325. DOI: 10.1101/344325.

Kispersky, Tilman J., Jonathan S. Caplan, and Eve Marder (2012) “Increase in Sodium Conductance Decreases Firing Rate and Gain in Model Neurons”. In: *Journal of Neuroscience* 32.32, pp. 10995–11004. DOI: 10.1523/JNEUROSCI.2045-12.2012.

Lin, Wei-Hsiang, Cengiz Günay, Richard Marley, Astrid A. Prinz, and Richard A. Baines (2012) “Activity-Dependent Alternative Splicing Increases Persistent Sodium Current and Promotes Seizure”. In: *Journal of Neuroscience* 32.21, pp. 7267–7277. DOI: 10.1523/JNEUROSCI.6042-11.2012.

Liu, Zheng, Jorge Golowasch, Eve Marder, and L. F. Abbott (1998) “A Model Neuron with Activity-Dependent Conductances Regulated by Multiple Calcium Sensors”. In: *Journal of Neuroscience* 18.7, pp. 2309–2320. DOI: 10.1523/JNEUROSCI.18-07-02309.1998.

Nadim, Farzan, Yair Manor, Nancy Kopell, and Eve Marder (1999) “Synaptic Depression Creates a Switch That Controls the Frequency of an Oscillatory Circuit”. In: *Proceedings of the National Academy of Sciences* 96.14, pp. 8206–8211. DOI: 10.1073/pnas.96.14.8206.

Nadim, Farzan, Yair Manor, Michael P. Nusbaum, and Eve Marder (1998) “Frequency Regulation of a Slow Rhythm by a Fast Periodic Input”. In: *Journal of Neuroscience* 18.13, pp. 5053–5067. DOI: 10.1523/JNEUROSCI.18-13-05053.1998.

O’Dowd, D. K. and R. W. Aldrich (1988) “Voltage-Clamp Analysis of Sodium Channels in Wild-Type and Mutant Drosophila Neurons”. In: *The Journal of Neuroscience: The Official Journal of the Society for Neuroscience* 8.10, pp. 3633–3643.

Prinz, Astrid A., Cyrus P. Billimoria, and Eve Marder (2003) “Alternative to Hand-Tuning Conductance-Based Models: Construction and Analysis of Databases of Model Neurons”. In: *Journal of Neurophysiology* 90.6, pp. 3998–4015. DOI: 10.1152/jn.00641.2003.

Prinz, Astrid A., Dirk Bucher, and Eve Marder (2004) "Similar Network Activity from Disparate Circuit Parameters". In: *Nature Neuroscience* 7.12, pp. 1345–1352. DOI: 10.1038/nn1352.

Rodriguez, Jason C., Dawn M. Blitz, and Michael P. Nusbaum (2013) "Convergent Rhythm Generation from Divergent Cellular Mechanisms". In: *The Journal of Neuroscience: The Official Journal of the Society for Neuroscience* 33.46, pp. 18047–18064. DOI: 10.1523/JNEUROSCI.3217-13.2013.

Sharp, A. A., M. B. O'Neil, L. F. Abbott, and E. Marder (1993) "Dynamic Clamp: Computer-Generated Conductances in Real Neurons". In: *Journal of Neurophysiology* 69.3, pp. 992–995. DOI: 10.1152/jn.1993.69.3.992.

Soto-Treviño, Cristina, Pascale Rabbah, Eve Marder, and Farzan Nadim (2005) "Computational Model of Electrically Coupled, Intrinsically Distinct Pacemaker Neurons". In: *Journal of Neurophysiology* 94.1, pp. 590–604. DOI: 10.1152/jn.00013.2005.

Soto-Treviño, Cristina, Kurt A. Thoroughman, Eve Marder, and L. F. Abbott (2001) "Activity-Dependent Modification of Inhibitory Synapses in Models of Rhythmic Neural Networks". In: *Nature Neuroscience* 4.3, pp. 297–303. DOI: 10.1038/85147.

Swensen, A. M. and E. Marder (2001) "Modulators with Convergent Cellular Actions Elicit Distinct Circuit Outputs". In: *The Journal of Neuroscience: The Official Journal of the Society for Neuroscience* 21.11, pp. 4050–4058.

Turrigiano, G., G. LeMasson, and E. Marder (1995) "Selective Regulation of Current Densities Underlies Spontaneous Changes in the Activity of Cultured Neurons". In: *The Journal of Neuroscience: The Official Journal of the Society for Neuroscience* 15 (5 Pt 1) pp. 3640–3652.

Wicher, Dieter, Christian Walther, and Carola Wicher (2001) "Non-Synaptic Ion Channels in Insects — Basic Properties of Currents and Their Modulation in Neurons and Skeletal Muscles". In: *Progress in Neurobiology* 64.5, pp. 431–525. DOI: 10.1016/S0301-0082(00)00066-6.
